# Effect of Astaxanthin on Activation of Autophagy and Inhibition of Apoptosis in *Helicobacter pylori*-Infected Gastric Epithelial Cell Line AGS

**DOI:** 10.3390/nu12061750

**Published:** 2020-06-11

**Authors:** Hanbit Lee, Joo Weon Lim, Hyeyoung Kim

**Affiliations:** Brain Korea 21 PLUS Project, Department of Food and Nutrition, College of Human Ecology, Yonsei University, Seoul 03722, Korea; beachmn@naver.com (H.L.); jwlim11@yonsei.ac.kr (J.W.L.)

**Keywords:** *Helicobacter pylori*, astaxanthin, autophagy, apoptosis, AMP-activated protein kinase

## Abstract

*Helicobacter pylori* (*H. pylori*) infection leads to the massive apoptosis of the gastric epithelial cells, causing gastric ulcers, gastritis, and gastric adenocarcinoma. Autophagy is a cellular recycling process that plays important roles in cell death decisions and can protect cells by preventing apoptosis. Upon the induction of autophagy, the level of the autophagy substrate p62 is reduced and the autophagy-related ratio of microtubule-associated proteins 1A/1B light chain 3B (LC3B)-II/LC3B-I is heightened. AMP-activated protein kinase (AMPK) and mammalian target of rapamycin (mTOR) are involved in the regulation of autophagy. Astaxanthin (AST) is a potent anti-oxidant that plays anti-inflammatory and anti-cancer roles in various cells. In the present study, we examined whether AST inhibits *H. pylori*-induced apoptosis through AMPK-mediated autophagy in the human gastric epithelial cell line AGS (adenocarcinoma gastric) in vitro. In this study, *H. pylori* induced apoptosis. Compound C, an AMPK inhibitor, enhanced the *H. pylori*-induced apoptosis of AGS cells. In contrast, metformin, an AMPK activator, suppressed *H. pylori*-induced apoptosis, showing that AMPK activation inhibits *H. pylori*-induced apoptosis. AST inhibited *H. pylori*-induced apoptosis by increasing the phosphorylation of AMPK and decreasing the phosphorylation of RAC-alpha serine/threonine-protein kinase (Akt) and mTOR in *H. pylori*-stimulated cells. The number of LC3B puncta in *H. pylori*-stimulated cells increased with AST. These results suggest that AST suppresses the *H. pylori*-induced apoptosis of AGS cells by inducing autophagy through the activation of AMPK and the downregulation of its downstream target, mTOR. In conclusion, AST may inhibit gastric diseases associated with *H. pylori* infection by increasing autophagy through the activation of the AMPK pathway.

## 1. Introduction

Gastric cancer is the fifth most common malignant tumor and the third leading cause of cancer death in the world [1]. Risk factors for gastric cancer include genetics, diet, lifestyle, alcohol intake, and smoking. However, *Helicobacter pylori* (*H. pylori*) infection is the main risk factor for gastric cancer [2]. *H. pylori* is a gram-negative microaerophilic bacterium that is found in the human gastric mucosal layer. It is a human pathogen that neutralizes the harsh environment of the stomach by secreting urease [3], and causes chronic inflammation, gastritis, gastric atrophy and gastric adenocarcinoma [4].

One of the pathogenic mechanisms of *H. pylori* infection is the apoptosis of gastric epithelial cells. Upon *H. pylori* infection, enzymes and cytotoxic proteins are secreted that damage the gastric mucosa. Increasing the apoptosis of epithelial cells due to damaged DNA stimulates cell proliferation to maintain tissue homeostasis. If hyperproliferation from the dysregulation of apoptosis is uncontrolled, neoplasia can occur [5,6]. Therefore, it is important to control the dysregulation of apoptosis to prevent uncontrolled tissue growth and cancer development.

Autophagy is an intracellular recycling system that maintains homeostasis in various pathological and physiological conditions, including starvation. Based on the manner in which cargo is delivered into the lysosome, three forms of autophagy have been identified. They are micro-autophagy, macro-autophagy, and chaperone-mediated autophagy (CMA). While each is morphologically distinct, all three culminate in the delivery of cargo to the lysosome for degradation and recycling [7]. Among three forms, macro-autophagy is best studied and reported in *H. pylori*-infected cells [8]. In *H. pylori*-infected cells, the virulence factor *vacA* induced autophagy to suppress toxin-induced cellular damage [8]. In addition, the upregulation of survival-related autophagy and downregulation of death-related apoptosis attenuates the inflammatory response to *H. pylori* infection [9]. In general, macro-autophagy participates in the disassembly of damaged organelles and the aggregation of proteins and pathogens by lysosomal fusion [10]. The autophagy cascade proceeds as follows: an autophagosome is formed, cargo is selected by p62, and the autophagosome is fused with a lysosome. Following degradation, the byproducts return to the cytosol to reprocess their macromolecular constituents and generate energy to maintain cell viability under unfavorable conditions, protecting the cells under stress conditions.

AMP-activated protein kinase (AMPK) consists of αβγ-subunits and fulfills a role as a regulator of energy levels in various stress conditions. The AMP/ATP ratio, Ca^2+^ levels, oxidative stress, and other factors can lead to the activation of AMPK, which is phosphorylated at Thr 172 in a catalytic α-subunit through the transfer of reversible phosphate groups by upstream kinases [11]. Another important signal molecule, Akt is a RAC-alpha serine/threonine-protein kinase that acts as an antagonist of AMPK [12]. In addition, AMPK is closely associated with the regulation of autophagy by activating Unc-51-like autophagy-activating kinase (ULK) 1 directly and indirectly. The direct pathway involves phosphorylating and activating ULK1, and the indirect pathway involves activating ULK1 by inhibiting the mammalian target of rapamycin (mTOR) [13,14]. Accordingly, activated AMPK generates autophagy for cell survival. AMPK was recently suggested as a therapeutic target for metabolic diseases, inflammation, lymphoma, and cancer [15,16,17]. Some studies have indicated that the activation of AMPK prevents apoptosis in response to *H. pylori* infection by inducing cytoprotective autophagy [16,18].

The autophagic process involves the formation and clearance of autophagosomes. Microtubule-associated proteins 1A/1B light chain 3 (LC3) is a major element of autophagosome formation and a biomarker for autophagy. It is a soluble protein that appears in two forms: LC3-I and LC3-II. LC3 is present in the LC3-I form in the cytosol. Upon the induction of autophagy, LC3-I is transformed into LC3-II by the attachment of phosphatidylethanolamine, which is attached to both the outer and inner membranes of an autophagosome [19,20]. An increase in LC3 puncta and autophagic vacuoles suggests the activation of autophagy. Therefore, LC3B-I and LC3B-II protein levels have been used as autophagy markers.

Astaxanthin is an orange-red colored carotenoid pigment found in algae, yeast, and aquatic animals and is used in the nutraceutical, cosmetics, food, and feed industries [21,22]. Chemically, astaxanthin has a long backbone and two ionone rings bound with hydroxyl and keto groups [18]. Because of the lipophilic and hydrophilic structure that allows it to penetrate cell membranes, its antioxidant capacity, and intracellular absorption capacity, astaxanthin is superior in many ways to other antioxidants [23,24,25]. Based on anti-oxidant, anti-inflammation, and anti-tumor effects, astaxanthin stands out as a beneficial compound without serious side effects [26]. Some studies have shown that astaxanthin reduces oxidative stress-induced DNA damage, suppresses apoptosis, and activates AMPK for energy production and tissue protection [27,28,29]. Since oxidative stress mediates apoptosis by increasing caspase-3 activity and apoptotic protein Bax and decreasing anti-apoptotic protein Bcl-2, astaxanthin is reported to suppress apoptosis in various ways by reducing reactive oxygen species (ROS) [30]. We showed that an antioxidant lycopene and a NADPH (nicotinamide adenine dinucleotide phosphate) oxidase inhibitor, diphenyleneiodonium, inhibit apoptotic cell death in *H. pylori*-infected cells [31,32]. Recently, we demonstrated that astaxanthin increases catalase expression by activating peroxisome proliferator-activated receptor-γ and reduces ROS in *H. pylori*-infected cells [33]. Therefore, astaxanthin may inhibit apoptosis by reducing oxidative stress in *H. pylori*-infected cells. However, it has not been established whether astaxanthin induces autophagy to inhibit apoptosis and prevents cell death in *H. pylori*-infected cells.

The aim of this study was to determine whether astaxanthin induces AMPK-mediated autophagy activation and inhibits apoptotic cell death in in *H. pylori*-stimulated AGS cells. Autophagy activation was determined by levels of mTOR, p-mTOR, AMPK, p-AMPK, Akt, p-Akt, ULK1, p-ULK1, p62, LC3 protein expression, and the number of LC3 puncta. Apoptosis was assessed with decreased cell viability and increased DNA fragmentation and caspase-3 activity.

## 2. Materials and Methods

### 2.1. Reagents

Astaxanthin was purchased from Sigma-Aldrich (St. Louis, MO, USA). It was dissolved in dimethyl sulfoxide (DMSO) (final concentration 10 mM) and stored under nitrogen gas at −80 °C. Before treatment, the astaxanthin stock solution was thawed and dissolved in fetal bovine serum to achieve the desired concentrations. The AMPK inhibitor compound C and AMPK activator metformin were purchased from Sigma-Aldrich and dissolved in DMSO (final concentration 10 mM) and phosphate-buffered saline (PBS; final concentration 1 M) respectively.

### 2.2. Cell Line and Culture Conditions

The human gastric epithelial cell line gastric adenocarcinoma (AGS, American Type Culture Collection (ATCC) CRL-1739) was purchased from ATCC (Rockville, MD, USA). The cells were grown in a complete medium consisting of RPMI (Roswell Park Memorial Institute) 1640 (Gibco, Grand Island, NY, USA) supplemented with 10% heat-inactivated fetal bovine serum (Gibco), 2 mM glutamine, 100 U/mL penicillin, and 100 µg/mL streptomycin (Sigma-Aldrich, St. Louis, MO, USA). The cells were cultured at 37 °C in a humidified atmosphere of 95% air and 5% CO_2_.

### 2.3. H. pylori Strain and Infection Procedure

*H. pylori*, strain NCTC (National Collection of Type Cultures) 11637 (*cag A*-positive, *vac A*-positive strain), was obtained from the American Type Culture Collection. The bacterium was inoculated onto chocolate agar plates (Becton Dickinson Microbiology Systems, Cockeysvile, MD, USA) at 37 °C under microaerophilic conditions using an anaerobic chamber (BBL Campy Pouch System, Becton Dickinson Microbiology Systems, Franklin Lakes, NJ, USA). AGS cells were cultured overnight to reach 80% confluency. Whole *H. pylori* was harvested from the chocolate agar plates, suspended in antibiotic-free RPMI 1640 medium supplemented with 10% fetal bovine serum, and then used to treat AGS cells. AGS cells were cultured in the presence of *H. pylori* at a cell to a *H. pylori* ratio of 1:50 (at a multiplicity of infection (MOI) of 50).

### 2.4. Experimental Protocol

To investigate the effects of astaxanthin, AGS cells were pre-treated with astaxanthin (25 or 50 nM) for 3 h and then stimulated with *H. pylori* for 24 h (for analysis of cell viability, caspase-3 activity, cytochrome C release, and DNA fragmentation) or 1 h (for analysis of mTOR, p-mTOR, AMPK, p-AMPK, Akt, p-Akt, ULK1, p-ULK1, p62, and LC3 protein expression). To determine the effects on autophagy, the cells were pre-treated with compound C (10 μM) or metformin (10 mM) for 1 h before *H. pylori* stimulation. Pretreatment time periods for astaxanthin, compound C, and metformin were adapted from previous studies [33,34,35,36,37].

To determine whether *H. pylori* changes the phosphorylation of mTOR and Akt with culture time, AGS cells were cultured in the presence of *H. pylori* at a cell to *H. pylori* ratio of 1:50 for 1, 2, and 6 h and levels of mTOR, p-mTOR, Akt, and p-Akt were assessed by Western blotting.

### 2.5. Measurement of Cell Viability

The cells were seeded at 3.5 × 10^4^ in a 24-well culture plate, cultured overnight, and then infected with *H. pylori* at the indicated ratio (cell to *H. pylori* ratios of 1:20, 1:50, and 1:100) for 24 h. Numbers of viable cells were determined using trypan blue exclusion assay (0.2%, trypan blue; Sigma).

### 2.6. Preparation of Cell Extracts

The cells were stimulated with *H. pylori*, harvested with trypsin–EDTA (ethylenediaminetetraacetic acid), and then pelleted by centrifugation at 1000× *g* for 5 min. The cell pellets were resuspended in lysis buffer containing 1 M Tris pH 7.4, 1 M NaCl, 1% nonidet P-40 (NP-40), 10% sodium deoxycholate, 10% sodium dodecyl sulfate (SDS), and commercial protease inhibitor complex (Complete; Roche, Mannheim, Germany), and lysed by drawing the cells into a 1-mL syringe with several rapid strokes. The mixture was then incubated on ice for 30 min and centrifuged at 10,000× *g* for 15 min. The supernatants were collected and used as whole cell extracts. Protein concentrations were determined by Bradford assay (Bio-Rad Laboratories, Herculues, CA, USA).

### 2.7. Assessment of DNA Fragmentation

DNA fragmentation was measured by quantifying cytoplasmic oligonucleosome-bound DNA fragments. The cells (3.5 × 10^4^ cells/well) in 24-well plates were lysed and centrifuged at 200× *g* for 10 min. Then, the supernatants were collected to detect DNA fragmentation. The amount of nucleosomes in the cell lysates was evaluated by sandwich enzyme-linked immunosorbent assay (Cell Death Detection ELISAPLUS kit; Roche). The relative amount of nucleosome-bound DNA in lysates was expressed as an enrichment factor, quantified from absorbance measurements of the samples at 405 nm.

### 2.8. Measurement of Caspase-3 Activity

Caspase-3 activity was determined using a fluorometric capsase-3 assay kit (Abcam, Cambridge, UK). Briefly, the cells stimulated with *H. pylori* were detached using trypsin–EDTA, centrifuged, and lysed with Caspase Cell Lysis Buffer (200 μL) on ice for 30 min. After centrifugation, the supernatant was used directly to determine enzyme activity. The cell lysate (50 μL) was incubated at 37 °C with caspase-3 substrate N-acetyl-Asp-Glu-Val-Asp-7-amino-4-trifluoromethyl-coumarin (Ac-DEVD-AFC) for 20 min. The amounts of released AFC were measured using a Victor 5 multilabel counter (PerkinElmer Life and Analytical Science, Boston, MA, USA) at 400 nm excitation and 505 nm emission wavelengths.

### 2.9. Immunofluorescence Staining for LC3B Puncta

To identify autophagosome production through LC3B puncta, cells were immunofluorescence stained. The cells were seeded on coverslips in 6-well plates, pre-treated with astaxanthin for 3 h, and then stimulated with *H. pylori* for 24 h. The medium was removed, and the cells were washed with PBS and fixed with 4% formaldehyde for 10 min at room temperature. The fixed cells were permeabilized with 0.1% Triton X-100 for 15 min at 15–25 °C. Then, the cells were blocked for 1 h in a blocking solution and incubated for 1 h with primary antibody against LC3B (L7543, Sigma-Aldrich). After washing with PBS, the cells were reacted with rhodamine-conjugated mouse anti-rabbit IgG antibody (sc-2492, Santa Cruz Biotechnology, Dallas, TX, USA) for 1 h. After the removal of the secondary antibody, the cells were washed with PBS and covered with the antifade medium Vectashield containing 4′,6-diamidino-2-phenylindole (DAPI). The preparations were stored for 30 min to allow saturation with DAPI. The cells stained with rhodamine-conjugated antibody were examined under a laser scanning confocal microscope (Zeiss LSM 880, Carl Zeiss Inc., Thornwood, NY, USA) and photographed. Each sample was analyzed using a threshold of >7 dots/cell. LC3B puncta-positive cells were quantified and expressed as % of cells with >7 LC3B puncta/total number of cells.

### 2.10. Acridine Orange (AO) Staining

To detect the characteristic structures of autophagy, acidic vesicular organelles were determined using acridine orange (AO) (Sigma-Aldrich). The cells were seeded on coverslips in 6-well plates, pre-treated with astaxanthin for 3 h, and then stimulated with *H. pylori* for 24 h. After removal of the medium, the cells were incubated in new medium containing 5 ug/mL AO for 20 min at 37 °C in the dark. They were then washed with PBS and then fixed with 4% formaldehyde for 20 min at room temperature. Next, the cells were rinsed twice with PBS and then immediately examined under a laser scanning confocal microscope (Zeiss LSM 880, Carl Zeiss Inc., Thornwood, NY, USA) and photographed. AO-positive cells were quantified and expressed as % of cells with AO-positive cells/total number of cells.

### 2.11. Western Blot Analysis

Whole cell extracts (10–40 μg) were loaded into lanes, separated by 7–13% SDS polyacrylamide gel electrophoresis under reducing conditions, and transferred onto nitrocellulose membranes (Amersham, Inc., Arlington Heights, IL, USA) by electroblotting. The transfer of protein was verified by reversible staining with Ponceau S. Membranes were blocked with 3% non-fat dry milk in TBS-T (Tris-buffered saline and 0.2% Tween 20) for 1 h at 15–25 °C. Antibodies against p-AMPK (#2531, Cell Signaling Technology, Danvers, MA, USA), AMPK (sc-74461, Santa Cruz Biotechnology), p-mTOR (#5536, Cell Signaling Technology), mTOR (#2972, Cell Signaling Technology), p-Akt (#9271, Cell Signaling Technology), Akt (#9272, Cell Signaling Technology), p-ULK1 (#5869, Cell Signaling Technology), ULK1 (sc-390904, Santa Cruz Biotechnology), LC3B (L7543, Sigma-Aldrich), p62 (sc-28359, Santa Cruz Biotechnology), cytochrome C (556432, BD Pharmingen, CA, USA), and actin (sc-1615, Santa Cruz Biotechnology) were diluted in TBS-T containing 3% dry milk and incubated with cells overnight at 4 °C. After washing with TBS-T, primary antibodies were detected with horseradish peroxidase-conjugated secondary antibodies (anti-mouse, anti- rabbit, anti-goat) visualized using an enhanced chemiluminescence (ECL) detection system (Santa Cruz Biotechnology) and exposure to BioMax MR film (Kodak, Rochester, NY, USA). Protein level was compared to that of the loading control actin. Intensity of each protein band was densitometrically quantified by using the software Image J (National Institutes of Health, USA). The densitometric analysis was performed for the ratio of p-mTOR/TOR, p-AMPK/AMPK, p-Akt/Akt, p-ULK1/ULK1, p62/actin, cytochrome C/actin, and LC3B-II/LC3B-1. Data are the mean ± S.E. of *n* = 3 independent experiments carried out on 4 samples for each experimental group. The ratio of control group (cells without *H. pylori* stimulation and without AST treatment) was set at 100%.

### 2.12. Statistical Analysis

All values are expressed as the mean ± standard error (SE) from three different experiments. For each experiment, the number of each group was 4 (*n* = 4 per each group). Analysis of variance (ANOVA), followed by Newman–Keul’s post hoc test, was used for the statistical analysis. A *p*-value of 0.05 or less was considered statistically significant.

## 3. Results

### 3.1. H. pylori-Induced AGS Cell Death, DNA Fragmentation, and Caspase-3 Activity Were Enhanced by an AMPK Inhibitor but Suppressed by an AMPK Activator

**Figure 1:**
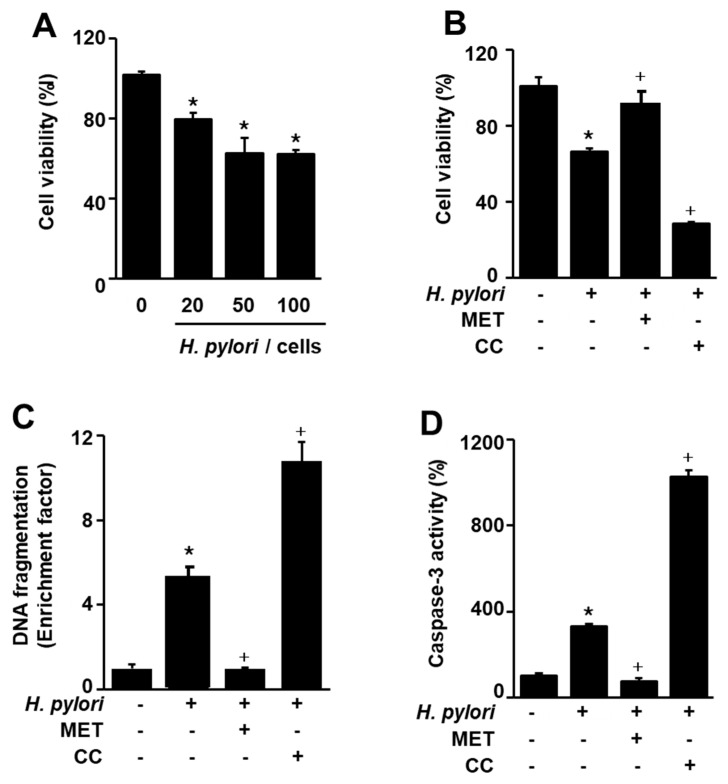
Effects of metformin and compound C on *Helicobacter pylori* (*H. pylori*)-induced cell death, DNA fragmentation, and caspase-3 activation in AGS cells. (**A**) The cells were stimulated at the indicated ratio of cells to *H. pylori* for 24 h. Cell viability of control group (cells without *H. pylori* stimulation) was set at 100%. (**B**–**D**) The cells were pre-treated with metformin (10 mM) and/or compound C (10 μM) for 1 h, and then stimulated with *H. pylori* (1:50) for 24 h. (**B**) Cell viability was measured using trypan blue exclusion assay. Cell viability of control group (cells without *H. pylori* stimulation and without any treatment) was set at 100%. (**C**) DNA fragmentation was assessed by the relative levels of nucleosome-bound DNA in cell lysates. DNA fragmentation of control group (cells without *H. pylori* stimulation and without any treatment) was set at 1. (**D**) Caspase-3 activity were measured using a caspase-3 activity assay kit. Caspase-3 activity of control group (cells without *H. pylori* stimulation and without any treatment) was set at 100%. * *p* < 0.05 vs. control (cells without *H. pylori* stimulation and without any treatment); + *p* < 0.05 vs. cells with *H. pylori* stimulation and without any treatment. Metformin (MET); compound C (CC).

To investigate whether *H. pylori* infection increases cell death, cell viability was measured at the indicated ratio of cells to *H. pylori* for 24 h. The number of viable cells was reduced by *H. pylori* infection at a cell to *H. pylori* ratio of 1:20, 1:50, and 1:100 (Figure 1A). Since *H. pylori*-induced cell death was similar between a cell/*H. pylori* ratio of 1:50 and 1:100, a cell/*H. pylori* ratio of 1:50 was used for further experiments on the effects of compound C, metformin, or astaxanthin.

To confirm that AMPK signaling is involved in *H. pylori*-induced cell death, the AMPK inhibitor compound C and AMPK activator metformin were used. *H. pylori*-induced cell death was enhanced by compound C, but was inhibited by metformin (Figure 1B). These results indicate that AMPK activation by metformin reduces *H. pylori*-induced cell death, while AMPK inhibition by compound C increases cell death in *H. pylori*-infected cells. Thus, the agents that activate AMPK may prevent *H. pylori*-induced cell death.

To examine apoptotic cell death associated with *H. pylori* infection, DNA fragmentation and caspase-3 activity assays were performed. *H. pylori* increased DNA fragmentation and caspase-3 activity, which are characteristics of apoptosis (Figure 1C,D). To confirm that AMPK signaling is involved in *H. pylori*-induced apoptotic cell death, compound C and metformin were used. *H. pylori*-induced DNA fragmentation and caspase-3 activity were enhanced by the AMPK inhibitor but inhibited by the AMPK activator (Figure 1C,D). These results indicated that *H. pylori*-induced apoptosis is mediated by AMPK signaling in AGS cells.

### 3.2. Astaxanthin Inhibits H. pylori-Induced Apoptosis in AGS Cells

**Figure 2:**
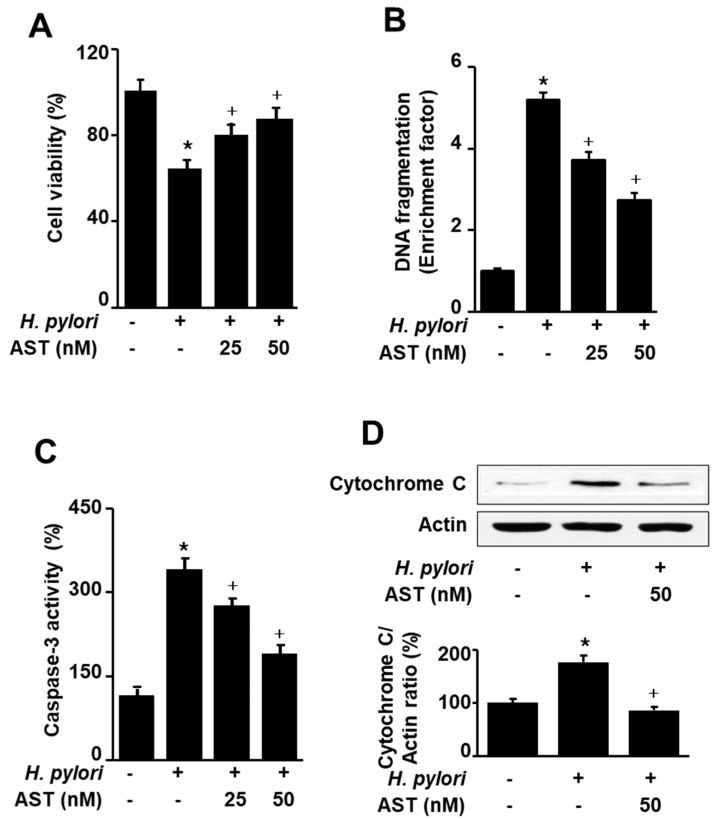
Effect of astaxanthin on *H. pylori*-induced cell death, DNA fragmentation, caspase-3 activation, and cytochrome C release in AGS cells. The cells were pre-treated with the indicated concentrations of astaxanthin for 3 h and then stimulated with *H. pylori* (cell to *H. pylori* ratio of 1:50) for 24 h. (**A**) Cell viability was measured using trypan blue exclusion assay. Cell viability of control group (cells without *H. pylori* stimulation) was set at 100%. (**B**) DNA fragmentation was assessed by relative levels of nucleosome-bound DNA in cell lysates. DNA fragmentation of control group (cells without *H. pylori* stimulation and without any treatment) was set at 1. (**C**) Caspase-3 activity was measured by caspase-3 activity assay kit. Caspase-3 activity of control group (cells without *H. pylori* stimulation and without any treatment) was set at 100%. (**D**) Cytochrome C release was determined with Western blotting. Actin was used as a loading control. The ratios of cytochrome C release/actin were determined from band densities of these proteins. Control (cells without *H. pylori* stimulation and without any treatment)) was set at 100. * *p* < 0.05 vs. control; + *p* < 0.05 vs. cells with *H. pylori* stimulation and without any treatment. Astaxanthin (AST).

To investigate the effect of astaxanthin on *H. pylori*-induced apoptotic cell death, AGS cells were stimulated with *H. pylori* in the presence or absence of astaxanthin. Apoptotic cell death was determined by viable cell numbers, DNA fragmentation, and caspase-3 activity assays, and cytochrome C release, respectively. Astaxanthin inhibited *H. pylori*-increased cell death, DNA fragmentation, caspase-3 activity, and cytochrome C release in AGS cells (Figure 2A–D). The results suggest that astaxanthin suppresses *H. pylori*-induced apoptosis in AGS cells.

### 3.3. Astaxanthin Activates AMPK Signaling and Induces Autophagy in H. pylori-Stimulated AGS Cells

**Figure 3:**
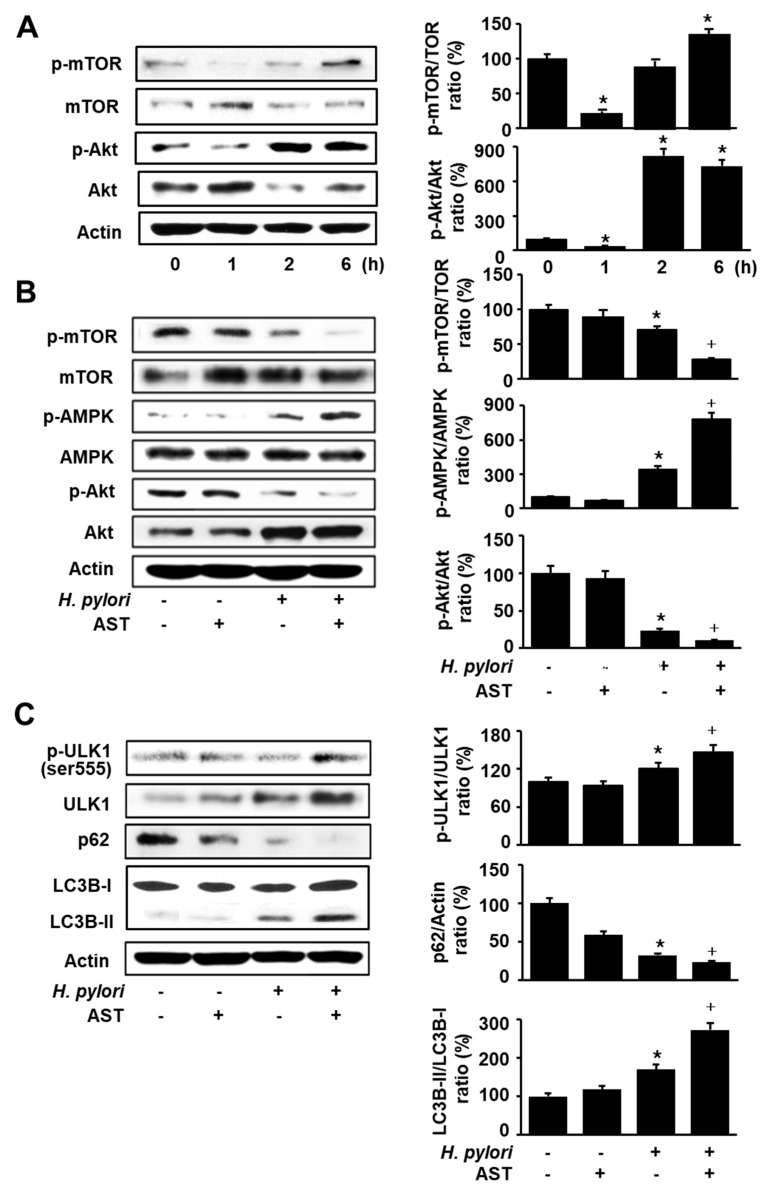
Effect of astaxanthin on phosphorylation of mammalian target of rapamycin (mTOR), Akt, and AMP-activated protein kinase (AMPK), expression of LC3-I/LC3-II and p62, and phosphorylation of Unc-51-like autophagy-activating kinase (ULK) 1 in *H. pylori*-stimulated AGS cells. (**A**) The cells were stimulated with *H. pylori* (cell to *H. pylori* ratio of 1:50) for the indicated time periods. * *p* < 0.05 vs. 0 h (**B**,**C**) The cells were pre-treated with 50 nM astaxanthin for 3 h and then stimulated with *H. pylori* (cell to *H. pylori* ratio of 1:50) for 1 h. The figure shows a representative Western blot for mTOR, p-mTOR, AMPK, p-AMPK, Akt, p-Akt, ULK1, p-ULK1, p62, LC3B-I, and LC3B-II protein levels and the densitometric analysis for the ratio of p-mTOR/TOR, p-AMPK/AMPK, p-Akt/Akt, p-ULK1/ULK1, p62/Actin, and LC3B-II/LC3B-1. Actin was used as a loading control. The data are expressed as arbitrary units obtained analyzing the bands by using the software Image J. Data are the mean ±S.E. of *n* = three independent experiments carried out on four samples for each experimental group. The ratio of control group was set at 100%. Control group represents the cells without *H. pylori* stimulation and without AST treatment. * *p* < 0.05 vs. control; + *p* < 0.05 vs. cells with *H. pylori* stimulation and without AST treatment. Astaxanthin (AST).

To determine the time-dependent changes in the phosphorylation of mTOR and Akt in *H. pylori*-stimulated cells, AGS cells were cultured in the presence of *H. pylori* for 1, 2, and 6 h and protein levels of mTOR, p-mTOR, Akt, and p-Akt were assessed (Figure 3A). *H. pylori* decreased the ratios of p-mTOR/mTOR and p-Akt/Akt at 1 h. Total forms of mTOR and Akt were increased, but p-mTOR and p-Akt were decreased by *H. pylori* infection after 1 h in culture. However, p-mTOR and p-Akt tended to be increased, while total forms of mTOR and Akt decreased up to 6 h. Thus, *H. pylori* increased the ratios of p-mTOR/mTOR and p-Akt/Akt at 2 h and 6 h of culture. For the further study, 1 h of culture was chosen to determine the effect of astaxanthin on AMPK signaling in *H. pylori*-infected cells.

To investigate whether astaxanthin activates the AMPK signaling pathway, western blotting was performed for total and phospho-specific forms of mTOR, Akt, and AMPK. Astaxanthin decreased the phosphorylation of Akt and mTOR in *H. pylori*-stimulated cells (Figure 3B). However, phosphorylation of AMPK is increased by astaxanthin in *H. pylori*-stimulated cells. These results suggest that astaxanthin activates the AMPK, but inhibits Akt and thus suppresses the mTOR signaling pathway in *H. pylori*-stimulated AGS cells. *H. pylori* infection slightly decreases the phosphorylation of Akt and mTOR, but slightly increases phosphorylation of AMPK in AGS cells.

To investigate the effect of astaxanthin on autophagy, we determined the protein levels of several autophagic markers (LC3B, p62, and ULK1) in *H. pylori*-stimulated AGS cells. In the state of autophagy, AMPK activates ULK1 and the LC3B-I cytosolic marker is transformed to the autophagosome component LC3B-II. p62 is an autophagy receptor and interacts with LC3-II and degraded by lysosomal proteases. As shown in Figure 3C, *H. pylori* infection slightly increased LC3B-II and slightly decreased p62 in AGS cells, which were augmented by astaxanthin treatment. Astaxanthin increased p-ULK in *H. pylori*-infected AGS cells.

**Figure 4:**
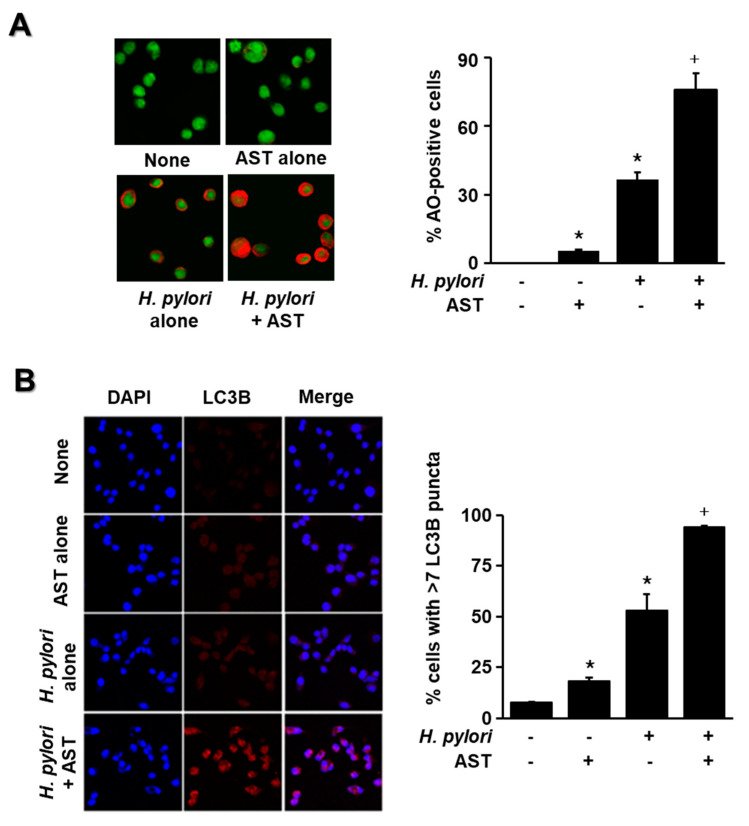
Effect of astaxanthin on autophagy activation in *H. pylori*-stimulated AGS cells. The cells were pre-treated with 50 nM astaxanthin for 3 h and then stimulated with *H. pylori* (cell to *H. pylori* ratio of 1:50) for 24 h. (**A**) Cells were stained with acridine orange (AO) dye and visualized under a confocal laser scanning microscope (left panel). AO-positive cells were quantified and expressed as % of cells with AO-positive cells/total number of cells (right panel). (**B**) The cells were stained with anti-LC3B antibody and rhodamine-labeled mouse anti-rabbit IgG antibody. Immunocytochemical staining for LC3B (red) and DNA counterstaining with DAPI (blue) are shown in the left panel. Each sample was analyzed using a threshold of >7 dots/cell. LC3B puncta-positive cells were quantified and expressed as % of cells with >7 LC3B puncta/total number of cells (right panel). * *p* < 0.05 vs. control (cells without *H. pylori* stimulation and without AST treatment); + *p* < 0.05 vs. cells with *H. pylori* stimulation and without AST treatment. Astaxanthin (AST).

The cells were stimulated with *H. pylori* in the presence or absence of astaxanthin, and the amount of fluorescence from LC3B and acidic vesicular organelles was measured by immunofluorescence and AO staining, respectively. These results indicate that *H. pylori* significantly increased the number of AO-positive cells and LC3B puncta in AGS cells (Figure 4A,B). Moreover, *H. pylori* stimulation with pre-treatment of astaxanthin increased AO-positive cells and LC3B puncta more than *H. pylori* stimulation without astaxanthin treatment (Figure 4A,B). These results suggest that astaxanthin activates autophagy in *H. pylori*-stimulated cells. Since astaxanthin acts as an autophagy activator in the present study, further study is useful to compare the effect of astaxanthin and an autophagy activator such as metformin as a positive control on the numbers of AO-positive cells and LC3B puncta-positive cells in *H. pylori*-infected cells.

## 4. Discussion

*H. pylori* is a major risk factor in the development of chronic gastritis and peptic ulcers, both of which can progress to gastric adenocarcinoma [38]. *H. pylori* infection contributes to increased rates of DNA damage and causes the apoptosis of gastric epithelial cells [39]. Based on previous studies, *H. pylori* is known to increase levels of apoptosis both in vivo and in vitro, playing a decisive function in pre-cancer transformation and development [5,40]. *H. pylori* leads to apoptosis through sequential caspase-8, -9, and -3 activity. In the present study, astaxanthin inhibited pro-apoptotic signals such as levels of caspase-3 activity and DNA fragmentation in *H. pylori*-infected cells. The results show that astaxanthin prevented *H. pylori*-induced cell death through its anti-apoptotic effects.

During carcinogenesis, autophagy acts as a tumor suppressor by decomposing damaged organelles and increasing genomic stability [41]. The virulence factor of *H. pylori*, *vacA*, induces autophagy that protects cells by restricting toxin-induced cell damage in short-term exposure [42]. Tsugawa et al. reported that *vacA* decreases the level of adenosine triphosphate, which promotes the AMPK activity related to autophagy [43].

AMPK controls downstream signaling to regulate cell survival, apoptosis, and autophagy, which is crucial to promote anti-apoptotic responses during *H. pylori* infection. In recent studies, treatment with an AMPK inhibitor (compound C) was shown to accelerate *H. pylori*-induced cell apoptosis, whereas AMPK activators (metformin and compound 13) were shown to mitigate apoptosis and enhance AMPK activation in *H. pylori*-stimulated cells [15,17,44]. In the present study, *H. pylori*-induced apoptosis was found to be associated with AMPK signaling in gastric epithelial AGS cells.

Autophagy is a protective mechanism that promotes cell survival by maintaining bioenergetic homeostasis [45,46]. Interaction between autophagy and apoptosis is complicated. Recent study suggests that autophagy is closely associated with apoptosis. Mostly, autophagy inhibits apoptosis, indicating that autophagy has an anti-apoptotic rather than pro-apoptotic function [47]. Cytoprotective autophagy can increase cell survival by preventing cell apoptosis [48]. Luo et al. [49] reported that Sirt1 increased autophagic flux and decreased apoptosis via AMPK activation to protect hypoxic H9C2 cardiomyocytes. The AMPK/mTOR pathway is closely connected to positive regulation of autophagy. AMPK signaling modulates phosphorylation of mTOR and ULK1, which are important kinases in autophagy mechanism. mTOR is a serine/threonine protein kinase that negatively regulates ULK1 phosphorylation either directly or indirectly. Dephosphorylated mTOR has been found to separate ULK1 from mTORC1, and phosphorylated ULK1 has been shown to augment autophagy [50]. Another autophagy-related pathway is the phosphatidylinositol-3-kinase (PI3K)/Akt/mTOR signaling pathway, which is involved in cell growth and proliferation and functions as a tumor promoter [51]. Activated Akt directly phosphorylates downstream mTOR, thereby inhibiting autophagy. Furthermore, Akt functions as a cellular antagonist of AMPK, which precludes the phosphorylation of AMPK at Thr 172 [52,53]. Some studies have shown that the regulation of the AMPK/Akt/mTOR pathway induces autophagy by upregulating AMPK and downregulating Akt [54,55]. In addition to the AMPK/Akt/mTOR pathway, we determined LC3B-I and LC3B-II protein levels, since an increase in LC3B puncta and autophagic vacuoles represents the activation of autophagy for the determination of *H. pylori*-induced autophagy of AGS cells.

Astaxanthin is a potent antioxidant known for its anti-inflammatory and anti-cancer effects. Astaxanthin reduces oxidative stress-associated diseases and mitochondrial dysfunction [30] and suppresses gastric inflammation through anti-oxidant effects in *H. pylori*-infected mice [56]. Since ROS increased caspase-3 activation and the ratio of Bax/Bcl-2, astaxanthin suppressed oxidative stress-mediated apoptosis in lung epithelial cells [57], myocardiocytes [58], and neroblastoma cells [27]. Thus, the antioxidant effect of astaxanthin may reduce apoptosis by inhibiting caspase-3 activation and DNA fragmentation in *H. pylori*-infected cells.

Some studies indicated that AMPK activation leads to significant anti-inflammatory effects through multiple downstream pathways in various cell types and inflammatory disease models [59,60]. Yang et al. [61] reported that astaxanthin significantly elevated AMPK levels, showing a greater effect on AMPK phosphorylation than other antioxidants tested.

In clinical practice, metformin has been used as an oral antihyperglycemic agent for the treatment and prevention of type 2 diabetes [62]. However, metformin has several adverse effects such as metformin-associated lactic acidosis that makes it less palatable to patients. Thus, the providers or doctors must consider the risks before prescribing it [63]. However, there are no significant side effects reported in human studies in which astaxanthin was administered [64,65]. Several studies reported that oral supplementation with astaxanthin in healthy human volunteers and patients with reflux oesophagitis significantly reduced oxidative stress, hyperlipidemia and biomarkers of inflammation [65,66,67]. Therefore, astaxanthin may be a useful drug as an AMPK activator when compared to metformin.

In animal studies, astaxanthin decreased lipid peroxidation [66], inflammation [68] and thrombosis [69]. Dietary supplementation of astaxanthin reduced exercise-induced increases in oxidative stress biomarkers such as 8-hydroxy-2′-deoxyguanosine and 4-hydroxy-2-nonenal-modified protein in both the cardiac and gastrocnemius muscles of mice [70]. The mice that were fed 0.08% astaxanthin had higher cardiac mitochondrial membrane potential and contractility index compared with control animals [71]. These studies showed that astaxanthin prevents oxidative stress-induced inflammation and mitochondrial dysfunction. Therefore, astaxanthin may inhibit apoptosis by preventing abnormal mitochondrial function in animals infected with *H. pylori*. Previously, we used animal models infected with *H. pylori* and demonstrated the inhibitory effect of Angelica keiskei [72] and Korean red ginseng extract [73] on the gastric inflammation of mice and Mongolian gerbils exposed to *H. pylori*. Therefore, further study should be performed to determine whether astaxanthin supplementation inhibits apoptosis and activates the autophagy of gastric mucosal tissues of the animals infected with *H. pylori.*

Apoptosis is triggered by two major pathways, an intrinsic pathway and an extrinsic pathway. Intrinsic apoptosis is induced by the increased permeability of the mitochondrial outer membrane. This permeability leads to cytochrome c release, apoptosome formation (cytochrome c /Apaf-1), and the processing of pro-caspase-9 to the mature form. The extrinsic apoptosis pathway is mediated by ligand/receptor binding, which results in the activation of caspase-8 [74]. Caspase-8 directly activates caspase-3 and Bid (BH3 interacting-domain death agonist), and tBid (truncated Bid) (as a result of the cleavage of Bid by caspase-8) stimulates cytochrome c release from the mitochondria. Cytochrome c forms apoptosomes and activates caspase-9, which, in turn, causes the maturation of caspase-3. The extrinsic and intrinsic pathways merge at caspase-3 and both ultimately result in cell death [75].

Chattopadhyay et al. [76] showed that *H. pylori*-induced apoptosis involves both the caspase-9-mediated mitochondrial pathway and the caspase-8-dependent extrinsic pathway. They demonstrated that the DNA repair activity of apurinic/apyrimidinic endonuclease-1 (APE-1) inhibits the mitochondrial pathway, while the acetylation function inhibits the extrinsic pathway during *H. pylori* infection. Thus, APE-1 differentially regulates the intrinsic and the extrinsic pathway of *H. pylori*-mediated gastric epithelial cell apoptosis. Lin et al. [77] demonstrated that human gastric epithelial cells sensitized to *H. pylori* confer susceptibility to tumor necrosis factor-related apoptosis-inducing ligand (TRAIL)-mediated apoptosis via the modulation of death receptor signaling. The induction of TRAIL sensitivity by *H. pylori* is dependent on the activation of caspase-8 and its downstream pathway.

Ravikuma et al. [78] suggested that autophagy can prevent the released cytochrome c from being able to form a functional apoptosome in the cytoplasm by the sequestration of damaged mitochondria. The study indicates that autophagy inhibits the intrinsic apoptosis pathway. In the present study, we found that an AMPK activator, metformin, inhibits *H. pylori*-induced apoptosis, while astaxanthin activates AMPK, which inhibits its downstream target, mTOR, thereby inducing autophagy. Astaxanthin activates ULK1 and induces autophagosome formation by increasing the interaction of p62 and LC3B-II in *H. pylori*-infected cells. Astaxanthin inhibited *H. pylori*-increased cell death, DNA fragmentation, caspase-3 activity, and cytochrome C release in AGS cells The results demonstrate that both metformin and astaxanthin may inhibit intrinsic apoptosis by activating autophagy, which prevents the release of cytochrome C from mitochondrial in *H. pylori*-infected cells. In contrast, an AMPK inhibitor, compound C, may increase *H. pylori*-induced apoptosis by the enhancement of the intrinsic apoptosis pathway. Further study is necessary to determine whether astaxanthin affects intrinsic apoptosis by assessing caspase-9 activity and extrinsic apoptosis by determining caspase-8 activity and TRAIL sensitivity in the cells infected with *H. pylori.*

Regarding the phosphorylation of mTOR, Akt, and AMPK in *H. pylori*-stimulated gastric tissues and cells, Xie et al. [79] studied the correlation between *H. pylori* infection and activation of the phosphatidylinositol-3-kinase/Akt (PI3K/Akt) pathway in the mucosal tissues of the patients with chronic atrophic gastritis and gastric cancer. Immunohistochemical and western blot analyses showed that Akt and p-Akt in *H. pylori*-positive gastric tissues were higher than those in *H pylori*-negative tissues. In vitro cell experiments revealed that *H. pylori* increased p-Akt in AGS cells at 6 h and 12 h. Sokolova et al. [80] demonstrated that infection with *H. pylori* led to the phosphorylation of Akt in AGS cells. Xu et al. [81] showed that p-Akt increased in MKN-28 cells incubated with *H. pylori* (ATCC 11,637) infection (MOI of 10, 50, 100, and 200) for 2 h.

In contrast, Kim et al. [82] demonstrated that *H. pylori* infection modulates host cell metabolism through *vacA*-dependent inhibition of mTOR. They suggested that *vacA*-dependent mitochondrial perturbations results in the inhibition of mTOR, which activates ULK1, a positive regulator of autophagy, increases LC3-II, and lead to subsequent autophagy. They compared the autophagy using AGS cells, HEK293T cells (human embryonic kidney), and NCI-N87 (human gastric epithelial, male), infected at MOI of 100 with either wild type *H. pylori* (ATCC 11,637, *cagA+, vacA+*) and VM02 (isogenic to 11,637 except to deletion of *vacA*). King et al. [83] similarly showed that *H. pylori* decreased Akt phosphorylation in AGS cells. They found that *H. pylori* induces the dephosphorylation of 3-phosphoinositide-dependent kinase-1 (PDK-1), a master kinase that regulates the phosphorylation of Akt in AGS cells.

In the present study, we used AGS cells infected with ATCC 1137 at a MOI of 50 for 1 h. We found that *H. pylori* decreased p-Akt and p-mTOR, while increasing total forms of mTOR and Akt, thus leading to a reduction in the ratios of p-mTOR/mTOR and p-Akt/Akt in AGS cells at 1 h of culture. The present findings were supported by the studies by Kim et al. [82] and King et al. [84]. They showed that *H. pylori* (*cagA+, vacA+* strain) decreased Akt phosphorylation and inhibited mTOR activation, which increased the ratio of p-ULK1/ULK1 and LC3-II/LC3-I and autophagy in AGS cells.

At 6 h of culture, we found that *H. pylori* increased p-mTOR and p-Akt, but decreased total forms of mTOR and Akt in AGS cells. Thus, *H. pylori* increased the ratios of p-mTOR/mTOR and p-Akt/Akt at 6 h of culture. Xie et al. [79], Sokolova et al. [80], and Xu et al. [81] similarly demonstrated these findings, showing that infection with *H. pylori* led to the phosphorylation of Akt in AGS cells. Therefore, depending on the culture time, AMPK signaling may be differentially regulated in *H. pylori*-stimulated gastric epithelial cells. Further study should be performed to investigate time-dependent changes in the autophagy process and autophagy-related signaling molecules, which may contribute to understanding of the pathologic mechanisms of acute and chronic infection of *H. pylori* in gastric tissues.

Regarding p62 level, Raju et al. [42] demonstrated the accumulation of p62, a marker of autophagy, in AGS cells treated with culture supernatants from *vacA+* *H. pylori* for 24 h. They found that prolonged exposure of AGS cells to *vacA+* disrupted the induction of autophagy because the cells lacked cathepsin D in autophagosomes. Thus, the loss of autophagy resulted in the accumulation of p62 in AGS cells. Lim et al. [85] showed that AGS cells infected with *H. pylori* (P1 strain) increased p62, which promotes the interaction of A20 with procaspase-8 and removes polyubiquitin chains from procaspase-8 in pathogen infection. They found that the accumulation of p62 was shown from 3 h of culture until 9 h of culture with *H. pylori*, but p62 was not changed at 1 h of culture in AGS cells. Raju et al. [42] used the direct treatment of culture supernatants from *vacA+* *H. pylori* for 24 h to AGS cells. Lim et al. [85] treated the *H. pylori* P1 strain for 3 h. In contrast, Tang et al. [86] demonstrated that *H. pylori* (strain 26695) induces p62 degradation and increases LC3B II in a time-dependent manner, which induces autophagy in AGS cells. This study supports the present results demonstrating that *H. pylori* decreased p62 and increased the levels of LC3B-II/LC3B-I in AGS cells at 1 h of culture.

Antioxidants such as α-tocopherol and delphinidin induced autophagy by antioxidant activities [83,87]. α-tocopherol stimulated autophagy and reduced desmin aggregation, determined by increased levels of LC3B-II, in muscle cells [83]. Delphinidin inhibited ROS-induced apoptosis and induced autophagy. Autophagy was assessed by the formation of LC3B-II and autophagosomes (LC3B puncta and AO staining) in human chondrocytes [87]. Therefore, antioxidant compounds may increase the number of AO-positive cells and LC3B puncta.

On the basis of results from these and many other studies, we hypothesized that astaxanthin is involved in AMPK-mediated autophagy induction in *H. pylori*-stimulated AGS cells. In this study, increased AMPK phosphorylation with astaxanthin pre-treatment was demonstrated by measuring protein expression levels. Immunofluorescence and AO staining showed that astaxanthin increased fluorescence from LC3B and acidic vesicular organelles, which suggests that autophagy was increased by astaxanthin.

In summary, astaxanthin increases the phosphorylation of AMPK, which inhibits mTOR activation. Since mTOR suppresses the phosphorylation of ULK1, astaxanthin activates ULK1 and thus induces autophagosome formation by increasing LC3B-II in *H. pylori*-infected cells.

This study demonstrates the inhibitory effect of astaxanthin on *H. pylori*-induced apoptosis in human gastric epithelial AGS cells through the upregulation of cytoprotective autophagy and the modulation of the AMPK pathway. Through this mechanism, astaxanthin protects against cell damage caused by *H. pylori* infection.

## 5. Conclusions

This study provides evidence of the protective effects of astaxanthin against the *H. pylori*-induced apoptosis of gastric epithelial cells. Astaxanthin activates AMPK and inhibits its downstream target mTOR, thereby inducing autophagy. Through this molecular mechanism, astaxanthin significantly suppresses *H. pylori*-induced apoptosis. Therefore, astaxanthin is effective in mitigating the gastric disease associated with *H. pylori* infection.
